# Many-body effects in an MXene Ti_2_CO_2_ monolayer modified by tensile strain: GW-BSE calculations†

**DOI:** 10.1039/c9na00632j

**Published:** 2020-05-06

**Authors:** Yi-min Ding, Xiaomin Nie, Huilong Dong, Nopporn Rujisamphan, Youyong Li

**Affiliations:** Institute of Functional Nano & Solf Materials (FUNSOM), Jiangsu Key Laboratory for Carbon-Based Functional Materials & Devices, Soochow University Suzhou Jiangsu 215123 China yyli@suda.edu.cn; School of Chemistry and Materials Engineering, Changshu Institute of Technology Changshu Jiangsu 215500 China; King Mongkut's University of Technology Thonburi (KMUTT) 126 Pracha Uthit Road, Bang Mod, Thung Khru Bangkok 10140 Thailand

## Abstract

MXenes, two-dimensional (2D) layered transition metal carbide/nitride materials with a lot of advantages including high carrier mobility, tunable band gap, favorable mechanical properties and excellent structural stability, have attracted research interest worldwide. It is imperative to accurately understand their electronic and optical properties. Here, the electronic and optical response properties of a Ti_2_CO_2_ monolayer, a typical member of MXenes, are investigated on the basis of first-principles calculations including many-body effects. Our results show that the pristine Ti_2_CO_2_ monolayer displays an indirect quasi-particle (QP) band gap of 1.32 eV with the conduction band minimum (CBM) located at the M point and valence band maximum (VBM) located at the Γ point. The optical band gap and binding energy of the first bright exciton are calculated to be 1.26 eV and 0.56 eV, respectively. Under biaxial tensile strains, the lowest unoccupied band at the Γ point shifts downward, while the lowest unoccupied band at the M point shifts upward. Then, a direct band gap appears at the Γ point in 6%-strained Ti_2_CO_2_. Moreover, the optical band gap and binding energy of the first bright exciton decrease continuously with the increase of the strain due to the increase of the lattice parameter and the expansion of the exciton wave function. More importantly, the absorbed photon flux of Ti_2_CO_2_ is calculated to be 1.76–1.67 mA cm^−2^ with the variation of the strain, suggesting good sunlight optical absorbance. Our work demonstrates that Ti_2_CO_2_, as well as other MXenes, hold untapped potential for photo-detection and photovoltaic applications.

## Abstract 2

Modified band offset, band gap and optical response of Ti_2_CO_2_ monolayer by tensile strain.

## Graphical abstract

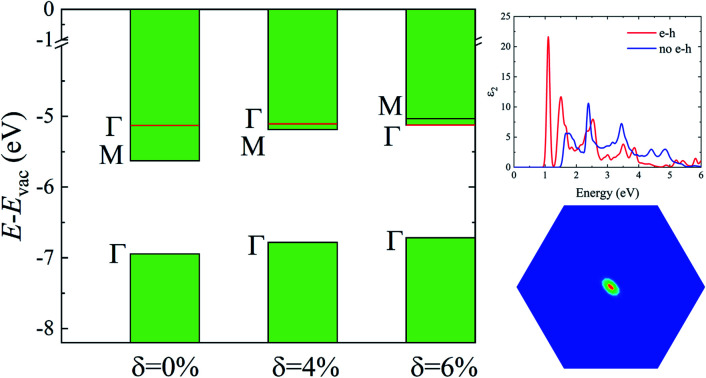

## Introduction

As a new member of the two-dimensional (2D) material family, MXenes, layered transition metal carbide/nitride materials, have aroused the interest of a great number of researchers, due to their advantages including high carrier mobility, tunable band gap, favorable mechanical properties and excellent structural stability.^1–4^ The first member of MXenes, Ti_3_C_2_T_*x*_ nanosheets, was produced by the room temperature exfoliation of Ti_3_AlC_2_ in hydrofluoric acid.^5^ After this, more than 20 kinds of MXenes have been successfully synthesized and dozens of hundreds of new MXenes have been theoretically simulated.^6^

In principle, 2D MXenes can be denoted as M_*n*+1_X_*n*_T_*x*_ (*n* = 1, 2 and 3), in which M is a transition metal (TM), X is nitrogen and/or carbon and T_*x*_ represents surface functional groups (*i.e.* –F, –O, or –OH). The flexible tunability of the elemental composition of MXenes leads to plenty of unique physical and chemical properties, which makes them suitable for numerous applications, such as electrodes of lithium/sodium-ion batteries, electrochemical catalysts, supercapacitors, hydrogen storage materials, molecular sensors, and antibacterial and bioimaging probe materials.^2,4,7^ Unfortunately, most MXenes have exhibited excellent metallic conductivity without an intrinsic bandgap, which limits their applications in laser diode (LD), field-effect transistor (FET), and light emitting diode (LED) devices.

Recently, great efforts have been made to find semiconducting MXenes. It is predicted that Sc_2_CF_2_, Sc_2_CO_2_, Ti_2_CO_2_, Zr_2_CO_2_ and Hf_2_CO_2_ have an indirect band gap, and only Sc_2_COH_2_ has a direct band gap of 0.45 eV.^8^ What is noteworthy is that the thermoelectric performance and elastic and optical properties of MXenes are highly dependent on the surface terminated groups T_*x*_.^9,10^ Recently, a two transition metal MXene Mo_2_TiC_2_O_2_ was found to be a 2D topological semiconductor with a band gap of 0.17 eV.^11^ For the first time, Lai *et al.* studied the transport properties of 2D Ti_2_CT_*x*_ FETs and obtained a high carrier mobility of 10^4^ cm^2^ V^−1^ s^−1^.^12^ They estimated an energy gap of 80 meV for Ti_2_CT_*x*_, where the functional group T_*x*_ is difficult to determine quantificationally due to the chemical exfoliation processes. Zhang *et al.* demonstrated that the carrier mobility of a Ti_2_CO_2_ monolayer reaches 10^2^ and 10^4^ cm^2^ V^−1^ s^−1^ for electrons and holes, respectively, and its indirect band gap is 0.91 eV on the basis of density functional theory calculations.^13^ Moreover, the band structures of Ti_2_CO_2_ can be tuned effectively by strain, and an indirect to direct band gap transition in Ti_2_CO_2_ was found under a tensile strain of 4%.^14^ With the increase of compressive strains, Ti_2_CO_2_ undergoes a transition from an indirect bandgap semiconductor to a metal.^15^ Accurate GW quasiparticle calculations show that the Ti_2_CO_2_ monolayer has an indirect bandgap of 1.15 eV, much larger than that found by density functional theory calculations.^16^ It is well known that excitonic effects have a crucial impact on the electronic and optical response of 2D materials caused by the incomplete dielectric screening and strong quantum confinement.^17,18^ However, there have been few reports on the excitonic properties of MXenes until now, which are vital for the development of MXene-based electronic and optical devices.

Herein, we aim to study the electronic and optical properties including excitonic effects of MXenes, taking the Ti_2_CO_2_ monolayer as an example, on the basis of many-body perturbation theory, *i.e.* Green's function (GW) plus Bethe–Salpeter equation (GW-BSE) calculations. The semiconducting characteristics of the Ti_2_CO_2_ monolayer have been confirmed not only by theoretical studies but also by experimental work,^12,16^ while the other semiconducting MXene materials have only been predicted by theoretical simulations.^8–10^ Our results reveal that the first bright exciton peak of the Ti_2_CO_2_ monolayer is located at 1.26 eV with an exciton binding energy as large as 0.54 eV. Under a biaxial tensile strain of 6%, the Ti_2_CO_2_ monolayer transforms into a direct bandgap semiconductor with the band gap increased to 1.59 eV at the Γ point. With the increase of strain, the excitation energy of the first bright exciton and binding energies decrease. And the absorbed photon flux of Ti_2_CO_2_ is calculated to be 1.76 mA cm^−2^, suggesting good sunlight optical absorbance.

## Computational methods and details

Firstly, the electronic ground states of the Ti_2_CO_2_ monolayer were calculated based on density functional theory (DFT) in the generalized gradient approximation (GGA) implemented in the QUANTUM ESPRESSO package.^19^ The optimized norm conserving Vanderbilt pseudopotentials with the Perdew–Burke–Ernzerhof (PBE) functional were employed with a plane-wave cutoff energy set at 100 Ry.^20^ The first Brillouin zone (BZ) was sampled using Monkhorst–Pack *k*-mesh of 15 × 15 × 1. The total energy and the atomic forces were converged within 10^−4^ eV and 0.01 eV Å^−1^, respectively, after structural optimization. The vacuum spacing was set at 15 Å to avoid any unnecessary interaction between adjacent layers. Furthermore, the magnitude of the in-plane strain was calculated using *δ* = (*a* −*a*_0_)/*a*_0_ × 100%, where *a*_0_ and *a* are the calculated pristine and strained lattice parameters. It should be pointed out that the structures of Ti_2_CO_2_ almost remain unchanged under the applied strains (from 0% to 6%). This simulation method of applying strains has been carried out on 2D materials and resulted in reasonable conclusions.^14,21^

As we all know, DFT in the formulation of Kohn and Sham is a ground-state theory in the form of an effective one-particle Schrödinger equation (the Kohn–Sham equation), which results in semiconductor bandgap underestimation problems and discrepancies between the calculated and experimental spectra.^22^ In order to overcome these problems, the excited states of real systems have to be calculated correctly. Many-body perturbation theory, a GW approach based on a set of Green's-function equations, has been successfully used to describe excited state properties and obtain more accurate band gaps.^23^ Furthermore, by solving the Bethe–Salpeter equation (BSE), the electron–hole interactions are well described, and the discrepancies between the calculated and experimental spectra could be eliminated.^24,25^ In brief, the GW-BSE approach, based on many-body perturbation theory, has been used widely to calculate the accurate band gap and absorption spectra. It can be used not only in simple systems but also in large systems such as Si supercells containing 1726 Si atoms.^26^ Considering the huge computational costs, researchers prefer to study simpler systems by GW-BSE approaches, such as small molecules, pristine 2D materials (InSe, MoS_2_, and black phosphorus) and graphene quantum dots.^18,27–29^

Our GW-BSE calculations were carried out within the BerkeleyGW code.^23,30,31^ The one-shot GW calculation was performed to obtain the quasi-particle (QP) bandstructure. The dynamical screening effects were included using the Hybertsen–Louie generalized plasmon pole (HL-GPP) model.^31^ Following the Nonuniform Neck Subsampling (NNS) method, the dielectric matrix was calculated on a 12 × 12 × 1 uniform *q* grid with an additional 10 *q*-points in the small-*q* region, which provides an efficient way to capture specific features of the dielectric matrix in 2D materials due to the electronic confinement.^32^ The dielectric matrix included plane-wave components up to 30 Ry, and about 1000 unoccupied states were included in the calculation of both the polarization and GW self-energy. Furthermore, the static remainder correction was added to the self-energy to speed up convergence with respect to summation over unoccupied states, and a truncated Coulomb interaction was used to prevent spurious interactions between periodic images.^33,34^ These parameters converged the QP gaps at Γ and M to better than 0.02 eV. The BSE matrix elements were calculated on a uniform 12 × 12 × 1 *k* grid and then interpolated to a 64 × 64 × 1 fine *k* grid using the Clustered Sampling Interpolation (CSI) technique.^32^ In CSI, BSE matrix elements are explicitly calculated for 4 additional *k* points in each cluster, sampled along the (100) direction. Six valence bands and three conduction bands were set to describe the excitons and to obtain the optical absorption spectra. These converged the BSE eigenvalues to better than 0.05 eV.

## Results and discussion

**Fig. 1:**
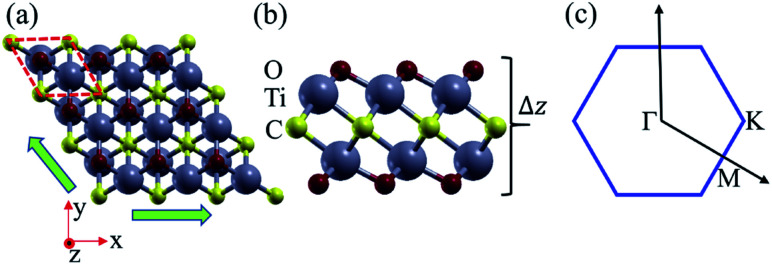
(a) Top and (b) side view of the crystal structure of the Ti_2_CO_2_ monolayer, and the shape of the Brillouin zone (c). Here the green arrows show the directions of the in-plane biaxial strains, and Δ*z* represents the out-plane thickness.

**Fig. 2:**
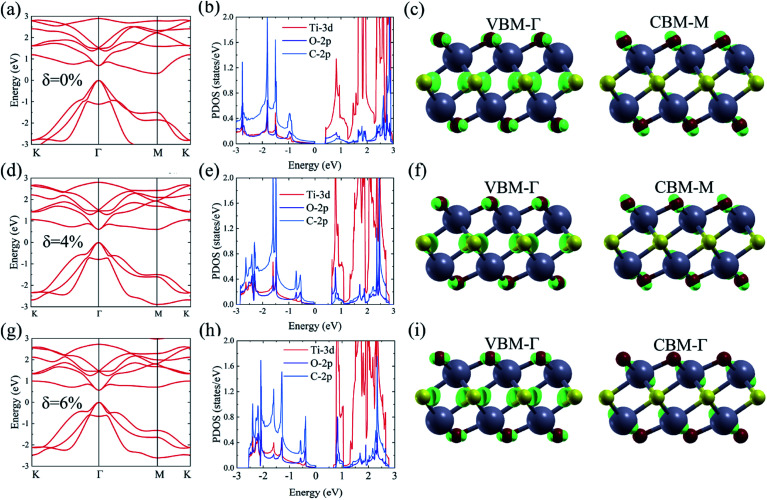
Calculated band structure, PDOS, and partial charge density of the CBM and VBM of the Ti_2_CO_2_ monolayer based on GGA calculations under strain, *δ* = 0% (a–c), 4% (d–f) and 6% (g–i), respectively.

As shown in Fig. 1, the Ti_2_CO_2_ monolayer consists of five atomic layers with a hexagonal unit cell, where the C layer is sandwiched between two Ti and two O layers. It has been confirmed to be the most stable structure of Ti_2_CO_2_ in previous studies.^8,35^ After structural relaxation, an equilibrium lattice constant of 3.03 Å for the pristine Ti_2_CO_2_ monolayer is obtained, which is consistent with that in previous theoretical studies (3.04/3.01 Å).^14,15^ Next, the band structures and projected density of states (PDOS) are calculated by the GGA method. From Fig. 2(a)–(c), we know that the Ti_2_CO_2_ monolayer has an indirect band gap of 0.32 eV, and the conduction band minimum (CBM) and valence band maximum (VBM) are located at the M and Γ point, respectively. Furthermore, the VBM of Ti_2_CO_2_ is mainly made up of C-2p and O-2p states, and the CBM is mainly made up of Ti-3d and O-2p states. Accordingly, the partial charge density of the VBM is distributed around C and O atoms, while the charge density of the VBM is distributed around Ti and O atoms. These results are consistent with previous calculations.^14^

**Fig. 3:**
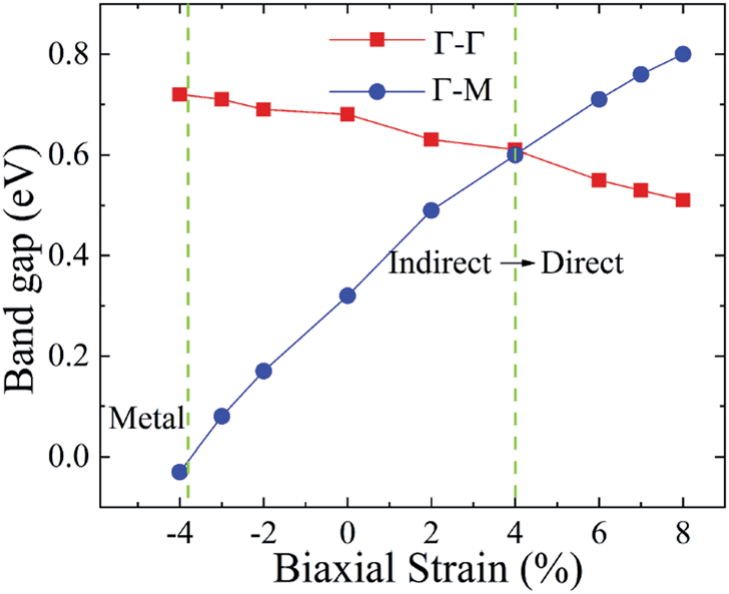
The Γ to Γ and Γ to M band gap values dependent on the various biaxial strains from −4% to 8%.

In general, indirect bandgap semiconductors are not efficient light emitters because a phonon with a high momentum is required to transfer an electron from the conduction band to the valence band.^36^ In order to improve the light emitting probability of the Ti_2_CO_2_ monolayer, we modified the electronic properties by applying in-plane biaxial strains. Fig. 3 shows the Γ to Γ and Γ to M band gap variations with the biaxial strains. With the increase of the strain from −4% to 8%, the Γ to Γ band gap values decrease continuously, and the Γ to M band gap values increase continuously. Two transition points are found. Under a compressive strain of *δ* = −4%, the direct band gap decreases to zero, leading to metallic properties in the Ti_2_CO_2_ monolayer. Under a tensile strain of *δ* = 4%, indirect–direct band gap transition is found. The direct band gap is very beneficial for light absorption and emission in semiconductors. Thus, we choose two strain values of *δ* = 4% and 6% before and after the indirect–direct bandgap transition point as representative situations and discuss their effects on the electronic and optical properties of the Ti_2_CO_2_ monolayer in detail.

Under a tensile strain of *δ* = 4%, the Ti_2_CO_2_ monolayer still exhibits an indirect band gap, which increases to 0.60 eV, with the VBM and CBM located at the Γ and M point, respectively, as presented in Fig. 2(d)–(f). Interestingly, Ti_2_CO_2_ becomes a direct bandgap semiconductor with both the CBM and VBM located at the Γ point under 6% strain, and the band gap decreases to 0.55 eV. Similar to that of pristine Ti_2_CO_2_, the VBM of strained Ti_2_CO_2_ is also made up of C-2p and O-2p states. Different from pristine Ti_2_CO_2_, the CBM of 6%-strained Ti_2_CO_2_ is only made up of the Ti-3d state with charge density distributed only around Ti atoms, as presented in Fig. 2(g)–(i). This is because the CBM has been shifted to the Γ point instead of the M point. As shown in Fig. 1(a), under in-plane tensile strains, the in-plane lattice is extended within the xy-plane. After the relaxation of atom positions, the material thickness Δ*z*, perpendicular to the xy-plane, decreases a little, which can be understood to be the Poisson effect. The distance between O and Ti/C atoms is reduced a little, which results in enhanced O–Ti/C interactions. Thus, the contribution of O in the CBM is significantly increased with the applied strain. Considering the bandgap underestimation problem in the usual DFT-GGA methods, we further carry out GW calculations to obtain accurate quasiparticle bandstructures of pristine and strained Ti_2_CO_2_ monolayers.

**Fig. 4:**
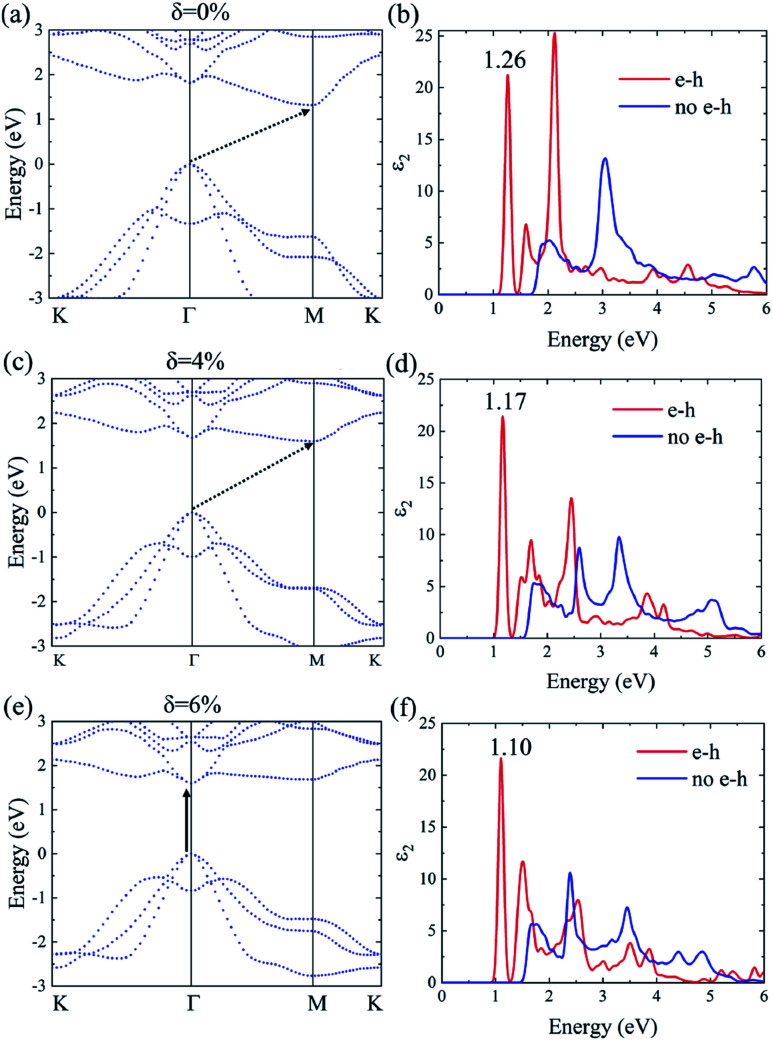
The G_0_W_0_ band structure and the imaginary part of the dielectric functions *ε*_2_ with and without e–h interactions of the Ti_2_CO_2_ monolayer under strain *δ* = 0% (a and b), 4% (c and d) and 6% (e and f), respectively.

The many-body effects, namely the electron–electron and electron–hole interactions for quasiparticles and optical excitations, can be well-described by the GW-BSE method, which gives results in good agreement with the experimental band gap and optical spectra, particularly in 2D materials.^18^ In GW approximation, the many-electron self-energy operator *Σ*, containing the effect of exchange and correlation among the electrons, was used to replace the exchange–correlation potential *V*_xc_ in DFT.^23^ The incorporation of many-body self-energy corrections results in more accurate QP energies and increased bandgap values. According to Fig. 4, the pristine Ti_2_CO_2_ monolayer still displays an indirect G_0_W_0_ band gap with the value increased to 1.32 eV (1 eV larger than that calculated by the DFT method), while the VBM and CBM are located at the Γ and M point, respectively. From the point of view of physics, the increase of the band gap is attributed to the incomplete dielectric screening and strong quantum confinement effect in suspended 2D materials.^37,38^ Under a tensile strain of *δ* = 4%, the Ti_2_CO_2_ monolayer also displays an indirect band gap of 1.59 eV, while the direct bandgap at Γ is 1.67 eV. For *δ* = 6%, Ti_2_CO_2_ transforms into a direct bandgap semiconductor with a band gap of 1.59 eV at Γ, lower than the indirect energy gap of 1.68 eV between Γ and M. In brief, Ti_2_CO_2_ can be transformed into a direct band gap semiconductor by tensile strain.

**Fig. 5:**
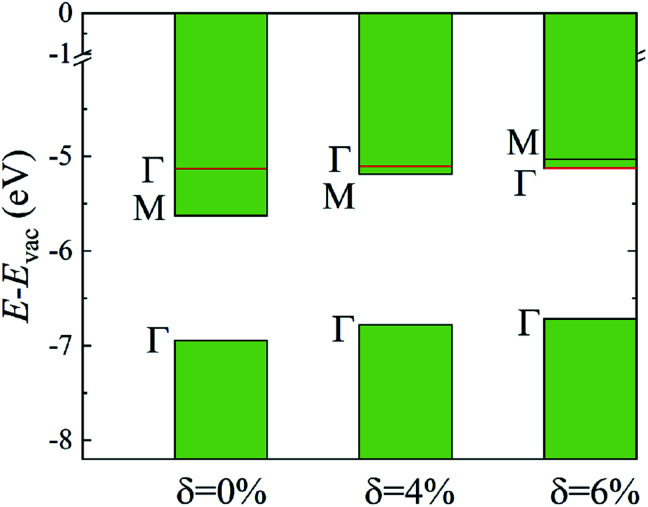
The calculated CBM and VBM alignment, relative to the vacuum level, for the different strain values. The shift of the energy level at Γ and M caused by strain is shown.

The evolutions of band gaps and band offsets of Ti_2_CO_2_ under strain are shown in Table 1 and Fig. 5. We can see that the energy level of the VBM at Γ moves up continuously under tensile strain. For the lowest conduction band, the energy level at Γ moves down, while the energy level at M moves up under strain. Finally, the energy level at Γ becomes lower than that at M, and thus a direct bandgap appears in Ti_2_CO_2_ at *δ* = 6%. In our calculations, the 6% tensile strain will lead to an obvious increase (0.18 Å) of the in-plane lattice constant and decrease of material thickness accordingly, which results in an upward shift of the lowest conduction band at M. The conduction-band inversion in the Ti_2_CO_2_ monolayer is thus induced by the tensile strain. Moreover, the GW method can accurately calculate the electron ionization and affinity potential and the energy difference of the VBM and CBM with respect to the vacuum level, respectively, as shown in Fig. 5. Under strain, both the affinity and ionization potential decrease from 5.63 to 5.12 eV and 6.95 to 6.71 eV, respectively. The decrease of the band gap and affinity and ionization potential of Ti_2_CO_2_ would result in enhanced photo-response.

**Table 1:** The calculated energy gaps *E*_g_ from Γ to Γ/M by GGA and GW methods and optical band gap *E*_opt_ and exciton binding energy *E*_b_ (in units of eV) of pristine and strained Ti_2_CO_2_ monolayers

	*E*_g_(GGA)		*E*_g_(GW)		*E*_opt_	*E*_b_
	Γ–Γ	Γ–M	Γ–Γ	Γ–M		
0%	0.68	0.32	1.82	1.32	1.26	0.56
4%	0.61	0.60	1.67	1.59	1.17	0.50
6%	0.55	0.71	1.59	1.68	1.10	0.49

**Fig. 6:**
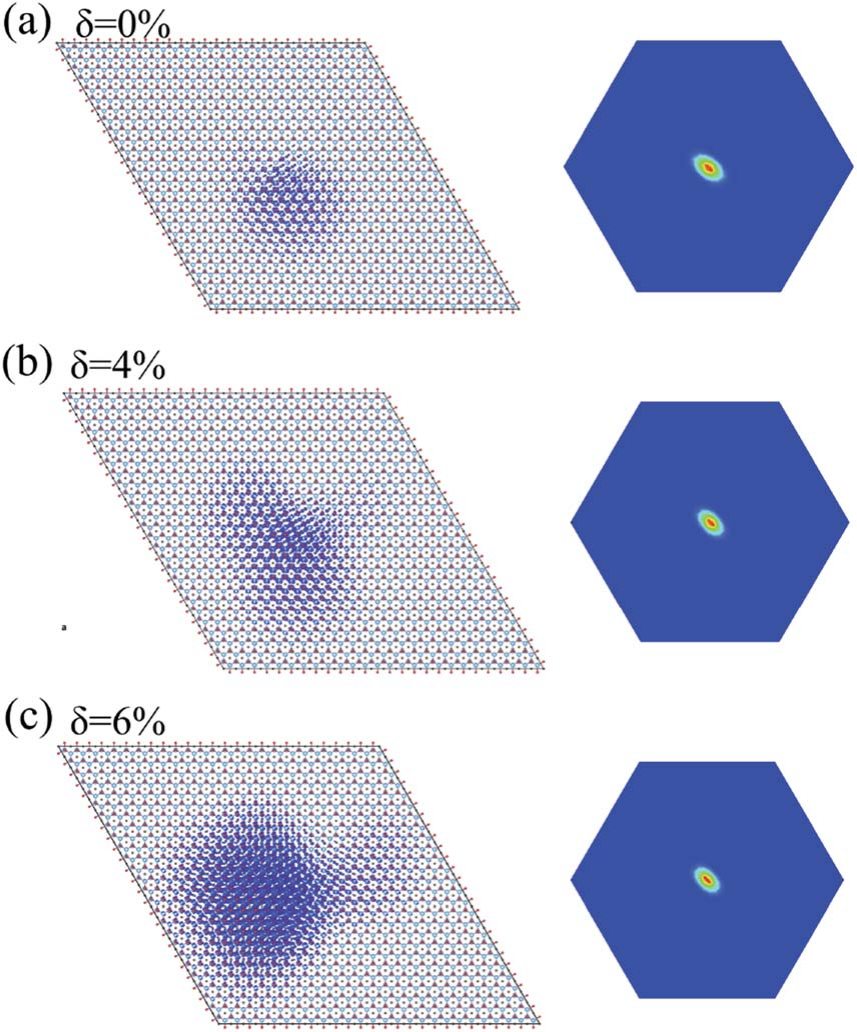
Exciton wave function for the lowest energy exciton in real space and *k*-space of the Ti_2_CO_2_ monolayer under strains of *δ* = 0% (a), 4% (b) and 6% (c), respectively.

The incorporation of the many-body effect has direct impacts not only on the correction of bandgaps but also on the optical spectra. The inclusion of electron–hole (e–h) interactions is essential for the calculation of optical spectra of 2D materials due to the reduced dimensionality and depressed screening. The enhanced excitonic effects caused by strong e–h interactions substantially reshape the optical spectra of 2D materials.^28^ Then, the imaginary part of the dielectric functions *ε*_2_, closely related to the absorbance spectra, is calculated with and without e–h interactions. From Fig. 4(b), (d) and (f), the absorption spectra calculated with e–h interactions have a redshift with respect to those calculated without e–h interactions. Thus, the main optical features are determined by bound excitonic states. The first absorption peak of the Ti_2_CO_2_ monolayer is located at 1.26 eV, which corresponds to the first bright exciton peak and is much smaller than the fundamental bandgap value from the calculation without e–h. Under strains of *δ* = 4% and 6%, the first bright exciton peak decreases to 1.17 and 1.10 eV, respectively, because of the reduction of the QP band gap at Γ. Fig. 6 shows the real-space and *k*-space distribution of the squared amplitude of first bright exciton wave functions. We can see that the main contributions for the first exciton peak are from the transitions between the highest valence and the lowest conduction bands close to the Γ point. In real space, the spatial distribution of the first bright exciton becomes more expanded under strain. The binding energy of an exciton is defined as the difference between the exciton's energy and the energy of the dominant band-to-band transition at Γ, where the lowest energy direct transition occurs.^39^ The binding energy of 0%, 4% and 6%-strained Ti_2_CO_2_ is 0.56, 0.50 and 0.49 eV, respectively. The decrease of binding energy is attributed to the extension of the exciton radius in real space caused by tensile strain, which results in reduced electron–hole interactions.

The first bright exciton peak of the Ti_2_CO_2_ monolayer is located at 1.26 eV (985 nm), which means Ti_2_CO_2_ has good potential for infrared detection and photovoltaic applications. In order to quantify the sunlight absorption of Ti_2_CO_2_, we further calculate the absorbed photon flux *J*_abs_ using^40,41^1
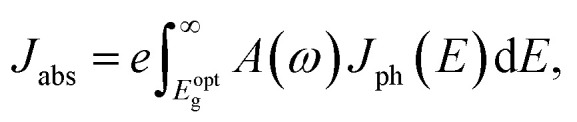
2
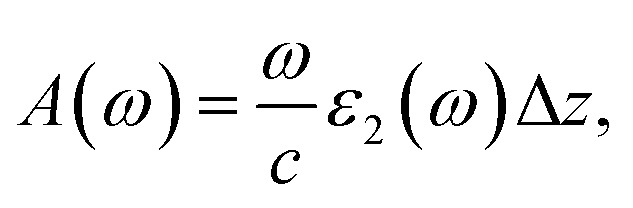
where *E* is the photon energy, *J*_ph_(*E*) denotes the incident photon flux (AM1.5G solar flux, units of photons per cm^2^ per s per eV), *c* is the speed of light and Δ*z* is the size of the simulated cell in the layer-normal direction. The absorbance *A*(*ω*) is defined as the fraction of photon energy (*E* = *ℏω*) adsorbed by the monolayer and is shown in the ESI.† According to Fig. S1,† under tensile strains of 4% and 6%, the first absorption peak red shifts, and the corresponding absorbance is increased by 60% from 6% to 10%, which can be attributed to the indirect–direct bandgap transition.

What's more, *J*_abs_ is expressed as the upper-limit short-circuit electrical current density (units of mA cm^−2^) in the ideal case where every photon is converted to a carrier extracted from light-absorbing materials. The absorbed photon flux *J*_abs_ is calculated to be 1.76, 1.74 and 1.67 mA cm^−2^ for 0%, 4% and 6%-strained Ti_2_CO_2_, respectively. The slight reduction of *J*_abs_ can be attributed to the compression of the out-plane thickness Δ*z* caused by the in-plane tensile strain. Most importantly, the calculated *J*_abs_ is much larger than that in 1 nm thick layers of Si (0.1), GaAs (0.3) and P3HT polymer (0.2),^40^ which suggests that Ti_2_CO_2_ shows great potential in photo-detection and photovoltaic applications.

## Conclusions

In conclusion, we have studied the electronic properties and optical response of the Ti_2_CO_2_ monolayer with and without tensile strain using first-principles calculations based on the GW-BSE formalism. Due to incomplete dielectric screening and quantum confinement in suspended 2D materials, the indirect QP band gap of the pristine Ti_2_CO_2_ monolayer is increased to 1.32 eV, 1 eV larger than that by GGA calculation. With the increase of tensile strain, the lowest conduction band at the Γ point shifts downward, while the lowest conduction band at the M point shifts upward. Then, a conduction-band inversion occurs, which results in a direct band gap at the Γ point in 6%-strained Ti_2_CO_2_. The optical band gap is 1.26, 1.17 and 1.10 eV, and the binding energy of the first bright exciton is 0.56, 0.50 and 0.49 eV for the 0%-, 4%- and 6%-strained Ti_2_CO_2_ monolayer, respectively. Moreover, the absorbed photon flux *J*_abs_ of Ti_2_CO_2_ without and with strain is calculated to be 1.76–1.67 mA cm^−2^, suggesting enhanced sunlight optical absorbance. Our study reveals the huge potential of Ti_2_CO_2_ in photo-detection and photovoltaics devices.

## Conflicts of interest

There are no conflicts to declare.

## Supplementary Material

NA-002-C9NA00632J-s001
