# Supplementary material for: Many-body effects in an MXene Ti2CO2 monolayer modified by tensile strain: GW-BSE calculations
Source: Nanoscale Adv. 2020 May 6;2(6):2471–7. doi: 10.1039/c9na00632j (PMC9417291; doi:10.1039/c9na00632j)
Supplement: NA-002-C9NA00632J-s001 [file NA-002-C9NA00632J-s001.pdf]

Electronic Supplementary Material (ESI) for Nanoscale Advances.
This journal is © The Royal Society of Chemistry 2020

Electronic Supplementary Information (ESI)

Many-body effects in MXene Ti_2CO_2 monolayer modified by tensile strain: GW-BSE calculations

Yi-min Ding,[a] Xiaomin Nie,[a] Huilong Dong,[b] Nopporn Rujisamphan,[c] Youyong Li[*a]

[a] Institute of Functional Nano & Sol f Materials (FUNSOM), Jiangsu Key Laboratory for Carbon-Based Functional Materials & Devices, Soochow University, Suzhou, Jiangsu 215123, China.

[b] School of Chemistry and Materials Engineering, Changshu Institute of Technology, Changshu, Jiangsu 215500, China

[c] King Mongkut's University of Technology Thonburi (KMUTT), 126 Pracha Uthit Road, Bang Mod, Thung Khru, Bangkok 10140, Thailand [*] Corresponding authors

E-mail: yyli@suda.edu.cn (Y. Li).

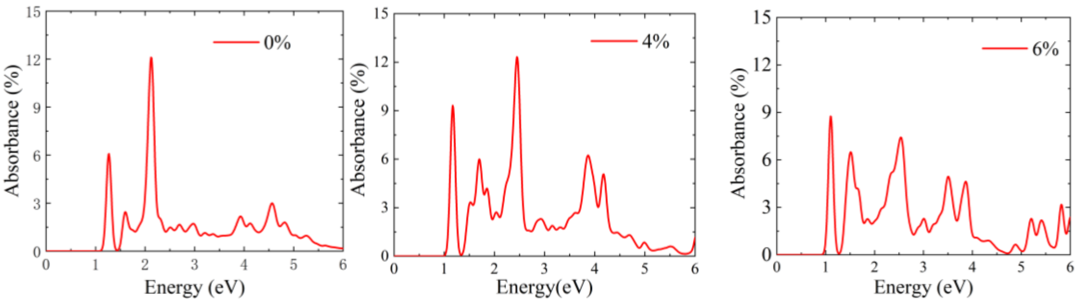

Fig. S1 Absorbance spectrum of Ti_2CO_2 under tensile strains of 0%, 4% and 6%.
